# Current trends for customized biomedical software tools

**DOI:** 10.6026/97320630013402

**Published:** 2017-12-31

**Authors:** Haseeb Ahmad Khan

**Affiliations:** 1 Department of Biochemistry, College of Science, King Saud University, Riyadh 11451, Saudi Arabia.

**Keywords:** Customized, biomedical software, tools

## Abstract

In the past, biomedical scientists were solely dependent on expensive commercial software packages for various applications. However, the advent of user-friendly programming languages and open source platforms has revolutionized the development of simple and efficient customized software tools for solving specific biomedical problems. Many of these tools are designed and developed by biomedical scientists independently or with the support of computer experts and often made freely available for the benefit of scientific community. The current trends for customized biomedical software tools are highlighted in this short review.

## Background

The enormous development in computer hardware and software technologies during the last few decades has revolutionized their application in biomedical sciences. The continuous surge in biological data requires more efficient computational tools for their analysis and interpretation. It was only a few decades back when a new branch named, Bioinformatics, was introduced to deal with computation and analysis of biological data. Within a short span of time, this branch flourished vastly and many academic institutions have now introduced undergraduate and graduate courses in bioinformatics. Bioinformatics is now considered as an interdisciplinary field related to biology, biochemistry, biotechnology, molecular biology and medicine. Biomedical scientists, if encountered with any computational complexity, tend to look for an appropriate software tool to solve the problem resulting in a new trend of developing customized software tools for dealing with specific biomedical problems. To highlight this trend, some useful software tools for wide range of applications are summarized in this short review. In fact, a large number of customized computer programs and packages have recently been published, only a selected number of representative studies are mentioned here.

A standalone application, FLIP, has been presented for analysis, organization, and illustration of structural data and molecular interactions for exploiting 3D-structures into simple 1D fingerprints. FLIP is free for academic use and provides a faster way to generate usable fingerprints for ligand and protein binding modes [1]. LiGRO is a freely available python-based graphical interface that was designed to overcome protein-ligand parameterization challenges by allowing the graphical control of GROMACS, ACPYPE and PLIP programs to be used together to fully perform and analyze the outputs of complex molecular dynamics simulations [2]. By allowing the calculation of linear interaction energies in a simple and quick fashion, LiGRO can be used in the drug-discovery pipeline to select compounds with a better protein-binding interaction profile. Yahyavi et al. [3] described a software program, VMD-SS, for the identification of secondary structure (SS) element and its trajectories during simulation for known structures available at the protein data bank. The program helps to calculate percentage SS, SS occurrence in each residue, percentage SS during simulation and percentage residues in all SS types during simulation. Saltbridges (SB) are specific electrostatic interactions that contribute to the overall stability of proteins. An efficient software tool, SBION, has been developed for rapid topological scan of a large number of proteins for extracting details on fraction of SB residues, chain specific intra-molecular SB, inter-molecular SB (protein-protein interactions), network SB and secondary structure distribution of SB residues [4].

Rodrigues-Luiz et al. [5] developed a software tool for identification of primers for multiple taxa (TipMT), which is a web application to search and design primers for genotyping based on genomic data. The tool identifies and targets single sequence repeats (SSR) or orthologous/taxa-specific genes for genotyping using multiplex PCR. GMATo (Genome-wide Microsatellite Analyzing Tool) is a freely available novel tool for SSR mining and statistical characterization in genomes at any size [6]. Che and Wang [7] have developed a computer program, Genomic Island Visualization (GIV), which displays the locations of genomic islands (GIs) in a genome, as well as the corresponding supportive feature information for GIs that can be related to special functionalities such as disease-causing GIs or pathogenicity islands. MethFinder is a tissue specific classifier program based on the frequency of novel sequence patterns across nine human tissues and is capable of discriminating methylation prone and methylation resistant CpG islands with an overall accuracy of 93% [8]. Orchid is a python based software package developed for the management, annotation, and machine learning of cancer mutations [9]. Building on technologies of parallel workflow execution, in-memory database storage, and machine learning analytics, orchid efficiently handles millions of mutations and hundreds of features in an easy-to-use manner. ENCoRE (Easy NGS-to-Gene CRISPR REsults) is a software tool that includes a simple graphical workflow, platform independence, local and fast multithreaded processing, data pre-processing and gene mapping with custom library import by easy manipulation of large raw next generation sequencing datasets. This software enables bench scientists with sensitive data or without access to informatics cores to rapidly interpret results from large-scale experiments resulting from pooled CRISPR/Cas9 library screens [10]. Maarala et al. [11] proposed ViraPipe, a scalable metagenome analysis pipeline that is able to analyze thousands of human micro-biomes in parallel in tolerable time with a throughput of 768 human samples in 210 minutes on a Spark computing cluster comprising 23 nodes and 1288 cores in total.

Lim et al. [12] proposed an R-based RNA sequence analysis pipeline called TRAPR that facilitates the statistical analysis and visualization of expression data. TRAPR provides various functions for data management, the filtering of low-quality data, normalization, transformation, statistical analysis, data visualization, and result visualization that allow researchers to build customized analysis pipelines. Khan [13, 14, 15] has developed Excel Add-ins for color-coded visualization, filtering and comparison of gene expression data and prediction of gene signatures. Muller et al. [16] demonstrated a customized imaging tool, AMIRA, to generate histologic sections of the prostate that directly correlate with needle-based optical coherence tomography pullback measurements. This technique will be crucial in validating the results of optical coherence tomography imaging studies with histology and will help improve the efficacy of this technique in cancer detection and staging in solid organs. A simple to use immunohistochemistry image analysis software, ImmunoMembrane, is freely available as a web application without requiring any download or installation. The software uses color de-convolution for stain separation and a customized algorithm for cell membrane segmentation while a quantitative score is generated according to the membrane staining intensity and completeness, for possible adoption of automated image analysis in clinical diagnostics [17]. Khan [18] has developed a simple and novel method for visualization of experimental gastric lesions by direct scanning of stomach samples and their quantitation by using computer-assisted image analysis.

For the benefit of organic and medicinal chemists, an algorithm has been described to identify functional groups in a molecule based on iterative marching through its atoms, resulting in identification of 3080 unique functional groups. The algorithm is relatively simple and therefore its implementation in any cheminformatics toolkit should be relatively easy [19]. Shoshi et al. [20] developed a new data warehouse KALIS, which is a webbased information system for health professionals and researchers and provides comprehensive knowledge and modules for risk analysis of drugs, which can contribute to minimizing prescribing errors and averting mortalities due to adverse drug interactions. Nasir et al. [21] developed a dataoriented bioinformatics workflow for efficient analysis of hundreds of thousands of glycopeptide MS/MS-spectra. Spicer et al. [22] reviewed the most widely used freely available software tools for metabolomics analysis, categorically based on their main functionality. CalcDose is a user-friendly Visual Basic tool that has been designed for dosage conversions between animals and humans, and is based on metabolic active mass measurements [23].

A freeware Add-in for Microsoft Excel, GInaFiT, aimed at bridging the gap between people developing predictive modeling approaches and end-users in the food industry not familiar with or not disposing over advanced non-linear regression analysis tools [24]. This tool is useful for testing nine different types of microbial survival models on user-specific experimental data relating the evolution of the microbial population with time. An algorithm, survival curves in Excel worksheet (SCEW) has been developed for easy creation of survival curves directly in Excel worksheets [25]. The advantages of this program are simple data input, minimal procedural steps and the creation of survival curves in the familiar confines of Excel. Khan [26] developed a computer program, CalcFisher, to solve the complexities involved in factorial computations for data analysis using Fisher's exact test. The complexity of factorial computations was greatly simplified by using logarithmic methodology as logbased computations are highly suitable for developing Visual Basic applications because they involve lesser number of operations and also keep the output of intermediate steps within the permissible range of Visual Basic. The operational simplicity and integrated report format of CalcFisher render a handy tool for performing Fisher's exact test [26]. A simple and user-friendly software (CalcNTCP) was developed for quick and accurate computation of normal tissue complication probability (NTCP), a key parameter to define the toxicity of radiation dose in cancer radiotherapy [27]. This software could be of potential application by assisting the clinicians in quick evaluation or optimization of the radiotherapy treatment plans. For testing visual field impairment, a free and open-source application (Specvis), written in Java programming language has been developed and tested on glaucomatous, retinitis pigmentosa and stroke patients [28]. The main advantages of Specvis over existing methods are its free availability, affordability, and reliability in parallel to high-cost solutions.

## Conclusion

The scope of bioinformatics has now extended form sequence analysis and molecular docking to more diverse applications such as drug safety, survival analysis, metabolomics, imaging, gene expression, gene editing and many more. A teamwork approach incorporating computer experts and biomedical scientists is important for building a strong hypothesis to develop an efficient software tool for end user expectations. However, with the advent of user-friendly programming languages, some biomedical scientists, even without any formal education in computer science but with self-learning of programming skills have been able to develop simple but highly useful software tools for specific biomedical applications. This trend is fascinating and expected to flourish in order to meet the complexities of exponentially emerging biological data.
